# Predictors of medication adherence in a large 1-year prospective cohort of individuals with schizophrenia: insights from the multicentric FACE-SZ dataset

**DOI:** 10.1038/s41398-023-02640-x

**Published:** 2023-11-07

**Authors:** David Misdrahi, Maud Dupuy, Yecodji Dansou, Laurent Boyer, Fabrice Berna, Delphine Capdevielle, Isabelle Chereau, Nathalie Coulon, Thierry D’Amato, Caroline Dubertret, Sylvain Leignier, Pierre Michel Llorca, Christophe Lançon, Jasmina Mallet, Christine Passerieux, Baptiste Pignon, Romain Rey, Franck Schürhoff, Joel Swendsen, Mathieu Urbach, Andrei Szöke, Ophélia Godin, Guillaume Fond, the FACE-SZ (FondaMental Academic Centers of Expertise for Schizophrenia) Group, M. Andre, C. Andrieu-Haller, B. Aouizerate, F. Berna, O. Blanc, E. Bourguignon, D. Capdevielle, I. Chereau-Boudet, J. Clauss-Kobayashi, N. Coulon, R. Dassing, J. M. Dorey, C. Dubertret, A. Esselin, G. Fond, F. Gabayet, M. Jarroir, D. Lacelle, M. Leboyer, S. Leignier, P. M. Llorca, J. Mallet, E. Metairie, T. Michel, D. Misdrahi, C. Passerieux, J. Petrucci, B. Pignon, P. Peri, C. Portalier, R. Rey, C. Roman, B. Schorr, F. Schürhoff, A. Szöke, A. Tessier, M. Urbach, G. Wachiche, A. Zinetti-Bertschy

**Affiliations:** 1 Fondation FondaMental, F-94010 Créteil, France; 2 University of Bordeaux, Aquitaine Institute for Cognitive and Integrative Neuroscience (CNRS UMR 5287-INCIA), Bordeaux, France; 3 Department of Adult Psychiatry, Charles Perrens Hospital, Bordeaux, France; 4 Université Paris-Est-Créteil (UPEC), AP-HP, Hôpitaux Universitaires “H. Mondor”, DMU IMPACT, INSERM, IMRB, Translational Neuropsychiatry, Bordeaux, France; 5 AP-HM, Aix-Marseille Univ, School of medicine - La Timone Medical Campus, EA 3279: CEReSS - Health Service Research and Quality of Life Center, 27 Boulevard Jean Moulin, 13005 Marseille, France; 6 Hôpitaux Universitaires de Strasbourg, Université de Strasbourg, INSERM U1114, Fédération de Médecine Translationnelle de Strasbourg, Strasbourg, France; 7 Service Universitaire de Psychiatrie Adulte, Hôpital la Colombière, CHRU Montpellier, Université Montpellier 1, Inserm 1061, Montpellier, France; 8 University of Clermont Auvergne, CMP-B CHU, CNRS, Clermont Auvergne INP, Institut Pascal, F-63000 Clermont-Ferrand, France; 9 Centre Référent de Réhabilitation Psychosociale et de Remédiation Cognitive (C3R), CH Alpes Isère, Bordeaux, France; 10 INSERM U1028, CNRS UMR5292, Centre de Recherche en Neurosciences de Lyon, Université Claude Bernard Lyon 1, Equipe PSYR2, Centre Hospitalier Le Vinatier, Pole Est, 95 bd Pinel, BP 30039, 69678 Bron Cedex, France; 11 AP-HP, Department of Psychiatry, Louis Mourier Hospital, Colombes, France; 12 Université Paris Cité, INSERM UMR1266, Institute of Psychiatry and Neuroscience of Paris, Paris, France; 13 Versailles Hospital, Department of Adult Psychiatry and Addictology, Centre Hospitalier de Versailles, 177 rue de Versailles, 78157 Le Chesnay, France; 14 DisAP-DevPsy-CESP, INSERM UMR1018, University of Paris-Saclay, University of Versailles Saint-Quentin-En-Yvelines, 94807 Villejuif, France; 15 University Department of Adult Psychiatry, La Colombiere Hospital, CHU Montpellier, University of Montpellier 1, Inserm 1061, Montpellier, France; 16 AP-HM, la Conception Hospital, Aix-Marseille Univ, School of medicine - La Timone Medical Campus, EA 3279: CEReSS - Health Service Research and Quality of Life Center, 27 Boulevard Jean Moulin, 13005 Marseille, France; 17 University Department of General Psychiatry, Charles Perrens Hospital, F-33076 Bordeaux, France; 18 Laboratory of Nutrition and Integrative Neurobiology (UMR INRA 1286), University of Bordeaux, Bordeaux, France; 19 Strasbourg University Hospital, University of Strasbourg, INSERM U1114, Federation of Translational Psychiatry, Strasbourg, France; 20 Clermont-Ferrand University Hospital, rue montalembert, Clermont-Ferrand Cedex 1, Bordeaux, France; 21 INSERM U955, Translational Psychiatry Team, DHU Pe‑PSY, Centre Expert Schizophrénie, Pôle de Psychiatrie et d’Addictologie des Hôpitaux Universitaires Henri Mondor, Paris Est University, 40 rue de Mesly, 94000 Créteil, France; 22 Schizophrenia Expert Center and Psychosocial Rehabilitation Reference Center, Alpes Isère Hospital, Grenoble, France; 23 INSERM, U1028; CNRS, UMR5292; University Lyon 1; Lyon Neuroscience Research Center, PSYR2 Team; le Vinatier Hospital, Schizophrenia Expert Centre, Lyon, F-69000 France; 24 Inserm UMR1266, Institute of Psychiatry and Neuroscience of Paris, University Paris Descartes, Paris, France; 25 Université Paris Diderot, Sorbonne Paris Cité, Faculté de médecine, Paris, France; 26 Department of Psychiatry (AP-HM), Sainte-Marguerite University Hospital, Marseille, France; 27 University of Bordeaux, CNRS UMR 5287-INCIA, Bordeaux, France

**Keywords:** Diseases, Schizophrenia

## Abstract

Schizophrenia is characterized by the most salient medication adherence problems among severe mental disorders, but limited prospective data are available to predict and improve adherence in this population. This investigation aims to identify predictors of medication adherence over a 1-year period in a large national cohort using clustering analysis. Outpatients were recruited from ten Schizophrenia Expert Centers and were evaluated with a day-long standardized battery including clinician and patient-rated medication adherence measures. A two-step cluster analysis and multivariate logistic regression were conducted to identify medication adherence profiles based on the Medication Adherence rating Scale (MARS) and baseline predictors. A total of 485 participants were included in the study and medication adherence was significantly improved at the 1-year follow-up. Higher depressive scores, lower insight, history of suicide attempt, younger age and alcohol use disorder were all associated with poorer adherence at 1 year. Among the 203 patients with initially poor adherence, 86 (42%) switched to good adherence at the 1-year follow-up, whereas 117 patients (58%) remained poorly adherent. Targeting younger patients with low insight, history of suicide, alcohol use disorder and depressive disorders should be prioritized through literacy and educational therapy programs. Adherence is a construct that can vary considerably from year to year in schizophrenia, and therefore may be amenable to interventions for its improvement. However, caution is also warranted as nearly one in five patients with initially good adherence experienced worsened adherence 1 year later.

## Introduction

Poor medication adherence is the primary cause of relapse in schizophrenia [1]. Seven decades of antipsychotic medication development (including the release of long-acting antipsychotics) have not been sufficient to address medication adherence issues in schizophrenia. Antipsychotics induce frequent side effects (e.g. impaired energy, motivation, and weight gain) that are the main reasons that patients withdraw from prescribed treatment regimes [2]. Cross-sectional studies have also revealed that poor medication adherence is associated to lack of insight (especially at the beginning of the illness), addictive behaviors, subjective negative attitudes toward medication, paranoid delusions resulting in altered capacity to consent to care, and cognitive impairment [3–9]. Most of these studies used exclusively clinical interviews to evaluate adherence which are known to overestimate medication adherence [10, 11]. To address this issue, the patient-reported Medication Adherence Rating Scale (MARS) was developed and validated in schizophrenia [12, 13]. Based on results from the MARS, we found in an initial cross-sectional study that younger age and low insight into illness were associated with poor medication adherence, and that depressive symptoms were also associated with poor adherence [2].

A frequent limitation of the studies published thus far is the over-reliance on cross-sectional designs, thus precluding patterns that may reveal causal relationships among correlated variables. Among the limited number of prospective investigations to have examined adherence, the combined data from the Clinical Antipsychotic Trials of Intervention Effectiveness (CATIE) and the European First Episode Schizophrenia Trial (EUFEST) demonstrated that substance use and impaired insight at baseline predicted poor adherence at 12 months [14, 15]. However, one potential bias of these studies was that they were unable to characterize “real-world” schizophrenia due to the hyper-selection process of randomized clinical trial studies. For this reason, prospective data are now needed to identify the predictors of adherence in unselected patients with schizophrenia so that effective and more generalizable interventions can be developed. The FondaMental Academic Centers of Expertise for schizophrenia cohort (FACE-SZ cohort) has been created to offer systematic, comprehensive, multi-dimensional and longitudinal assessments of cases, leading to therapeutic recommendations in the philosophy of precision medicine, and without strict selection criteria [16, 17]. The aim of this longitudinal study was to identify, by a clustering analysis from the MARS, predictors of poor medication adherence in this outpatient population at 1-year follow-up.

## Methods

### Recruitment and population

The FondaMental Academic Centers of Expertise for Schizophrenia (FACE-SZ) cohort was developed from the French national network of 10 Schizophrenia Expert Centers established for scientific cooperation by the FondaMental Foundation (www.fondation-fondamental.org), and in the goal of creating a platform linking healthcare and research. Outpatients aged 16 years or older with a DSM IV-TR diagnosis of schizophrenia or schizoaffective disorder were consecutively recruited for inclusion in the cohort. All study participants were referred by their general practitioner or psychiatrist and, contrary to common cohort methodology, only those who participated in the baseline and second visit as well as completed a MARS scale were included in the present study.

### Study design

The Expert Centers offer nation-wide access for all community-dwelling patients with schizophrenia in order to avoid biases associated with clinical trials [16, 17]. Their aim is to provide reliable, systematic, and standardized clinician-rated and patient-reported multi-dimensional assessments. A report with personalized recommendations for pharmacological, psychosocial and lifestyle interventions were provided at the end of the evaluation to the patients and the referring clinicians.

### Data collection

#### Medication adherence assessment

Medication adherence was evaluated using the patient-reported MARS questionnaire validated in schizophrenia [13, 18]. The sum of the 10 items yields a final score ranking from 0 (poorest adherence to treatment) to 10 (best adherence to treatment). The initial principal-components analysis revealed three underlying factors [12]. The first factor included the four first items and was related to “medication adherence behavior.” The second factor included the subsequent four items and represented the “subjects’ attitudes toward taking medication.” The remaining two items composed the third factor and represented “subjective negative side effects.” The Brief Adherence Rating Scale (BARS) which is a clinician-rated tool used to evaluate patient medication adherence during the last month was added to compare clinician assessment from the MARS self-rated adherence. Three items on adherence behavior (patient knowledge of the number of prescribed doses, number of days with less treatment taken, and no treatment taken during the last month) provide a guide for the clinician to complete a visual analog rating scale to assess overall medication adherence (0–100%) [19].

#### Sociodemographic and clinical variables

The following demographic and clinical variables at baseline were recorded: sex (binary variable), age (years), diagnosis (schizophrenia or schizoaffective as a binary variable), age of first psychotic episode (years), and illness duration (years). Psychotic symptomatology was assessed using the 5-factors Positive And Negative Syndrome Scale (PANSS, a continuous measure), [20, 21] and insight was measured using the Birchwood self-report Insight Scale for psychosis (BIS, continuous) that includes 3 subscores (illness awareness, symptoms awareness and perceived need for treatment) [22]. Lifetime history of suicide attempt, and lifetime history alcohol and cannabis use disorders (according to DSM V criteria) were reported as binary variables. Body Mass Index (BMI) was calculated at the expert center by a trained nurse.

Current psychotropic drugs were reported as binary variables: antipsychotic classes, clozapine, long-acting antipsychotic, chlorpromazine equivalent doses (CPZeq calculated according to the minimum effective dose method [23]), antidepressant, benzodiazepine, and total number of psychotropic treatments. Treatment side effects were measured using the Abnormal Involuntary Movements Scale (AIMS) [24] for tardive dyskinesia, the Barnes Akathisia Scale (BAS) [25] for drug-induced akathisia, and the Simpson and Angus Rating Scale (SARS) [26] for extrapyramidal side effects.

### Statistical analyses

Paired samples *T* tests and Wilcoxon signed-rank tests were used to assess difference in the mean MARS total score (and the three MARS mean subscores, respectively) between baseline and 1-year follow-up. The MARS items analysis at 1-year follow-up was completed by a two-step cluster analysis based on hierarchical clustering. The optimal number of clusters given the input variables was automatically selected according to the Akaike Information Criterion (AIC), which was used to identify latent types of attitude structures and to report behaviors in the individual patterns of responses to the 10 dichotomous items of the MARS. Response patterns of the two adherence clusters retained and membership probabilities were calculated from the estimated conditional response probabilities of the MARS items. A graphical representation was generated through cluster plot analysis (Supplementary Fig. S1).

To evaluate whether the identified clusters at the 1-year follow-up differed in socio-demographics and clinical data collected at baseline, comparisons were performed using Student’s *T* test or Wilcoxon signed-rank test for continuous variables (after examination for normal distribution) and chi-square tests for categorical variables. We used multivariate logistic regression to estimate odds ratios (ORs) to ascertain the effects of significant variables identified by univariate analyses between the 2 clusters, adjusting for the potential confounders defined by *p* value ≤0.20 in univariate analysis (Age, PANSS positive, PANSS negative, excitation, depressive and disorganization subscores, lifetime history of suicide attempts, alcohol use disorder, cannabis use disorder, Birchwood subscores, and BMI). A *p* value of <0.2 was chosen for covariates to capture a broader range of a potential large medication adherence predictors in the analysis. This threshold allows for the inclusion of variables that may have a modest association with medication adherence which is known to be multidetermined. The final models included OR and 95% confidence intervals (95% CI). To explore variables associated with the transition from one cluster at baseline to another at the 1-year follow-up, univariate and multivariable analyses were performed using the same method as detailed above.

To assess if the results could be linked to attrition bias, a sensitivity analysis was performed using an inverse probability-of-censoring weighting method. We calculated the probability of remaining in the study based on observed variables associated with loss to follow-up with *p* value ≤0.20 (Sex, PANSS subscores, Insight subscores, medication adherence (MARS), BMI, lifetime alcohol use disorder, extrapyramidal symptoms, first generation antipsychotics, second generation antipsychotics, antidepressants, number of psychotropic medications and long-acting antipsychotic administration) and multivariate analysis was weighted by the inverse of these probabilities. The statistical significance level was set at *p* < 0.05 for a two-sided test. All analyses were performed using R version 4.0.3 (R foundation).

### Ethical considerations

The study was carried out in accordance with ethical principles for medical research involving humans (WMA, Declaration of Helsinki). The assessment protocol was approved by the relevant ethical review board (CPP-Ile de France IX; January 18, 2010). The details of the cohort design and rationale have been presented in a previous publication [17]. A web-based application, e-Schizo^©^, was developed to collect evaluation data for clinical monitoring and research purposes. Access to this system is carefully regulated and approval was obtained from the ethical committee as well as the national committee in charge of the safety of computerized databases (CNIL). A non-opposition form was signed by participants according to French law.

## Results

### Sample characteristics

Analyses were performed on the 485 patients who completed a MARS evaluation at 1 year after inclusion. They were 376 (77.5%) men, mean aged 32.1 years (SD = 10.1) with a mean age at illness onset of 21.7 years (SD = 6.7) and mean illness duration of 10.3 years (SD = 8.2). The sociodemographic and clinical characteristics of the sample are presented in Table 1.

**Table 1:** Sample characteristics and predictors of adherence at 1 year.

	Adherence to medication at 12 months of visit				Multivariable analysis		
	Whole sample	Cluster 1 “Poor adherence” *N* = 170 (35.1%)	Cluster 2 “Good adherence” *N* = 315 (64.9%)	Univariate analysis			
				*p* value	aOR^a^	95% CI	*p* value
Sociodemographic characteristics							
Sex, male, *n* (%)	376 (77.5)	136 (80.0)	240 (76.2)	0.338			
** Age, mean (sd)**	**32.11 (10.1)**	**30.97 (8.8)**	**32.73 (10.6)**	**0.051**	**0.97**	**0.94–0.99**	**0.043**
Diagnosis, schizophrenia (vs schizoaffective disorder)	367 (75.7)	125 (73.5)	242 (76.8)	0.419			
Clinical variables							
Age at illness onset, mean (sd)	21.73 (6.7)	21.34 (6.6)	21.95 (6.7)	0.339			
Illness duration (years), mean (sd)	10.34 (8.2)	9.77 (7.2)	10.65 (8.7)	0.632			
** PANSS total score, mean (sd)**	**69.52 (17.9)**	**72.77 (18.6)**	**67.71 (17.4)**	**0.004**	1.01	0.98–1.02	
** PANSS positive score, mean (sd)**	**8.25 (3.9)**	**8.98 (3.7)**	**7.85 (3.9)**	**<0.001**	**1.07**	**1.00–1.15**	**0.024**
** PANSS negative score, mean (sd)**	**16.06 (6.7)**	**16.83 (6.7)**	**15.64 (6.7)**	**0.044**	1.02	0.97–1.06	0.323
** PANSS Excitation score, mean (sd)**	**5.39 (2.02)**	**5.72 (2.2)**	**5.21 (1.9)**	**0.003**	0.97	0.85–1.11	0.474
** PANSS Depressive score, mean (sd)**	**6.82 (3.0)**	**7.65 (3.2)**	**6.37 (2.8)**	**<0.001**	**1.19**	**1.09–1.31**	**<0.001**
PANSS Disorganized score mean (sd)	7.37 (3.3)	7.62(3.3)	7.24 (3.4)	0.171	0.96	0.87–1.05	0.370
** Insight Birchwood total score, mean (sd)**	**8.96 (2.84)**	**8.48 (2.9)**	**9.22 (2.8)**	**0.003**	**0.87**	**0.79–0.95**	
Insight illness awareness Birchwood subscore, mean (sd)	2.70 (1.37)	2.58 (1.4)	2.77 (1.4)	0.139	0.97	0.77–1.23	0.984
Insight symptoms awareness Birchwood subscore, mean (sd)	3.01 (1.20)	2.87 (1.3)	3.09 (1.2)	0.073	0.99	0.78–1.25	0.993
** Insight needs for treatment subscore, mean (sd)**	**3.25 (0.96)**	**3.01 (1.1)**	**3.38 (0.9)**	**<0.001**	**0.58**	**0.43–0.78**	**<0.001**
** Lifetime history of suicide attempt** (%)	**139 (29.3)**	**62 (37.1)**	**77 (25.1)**	**0.006**	**1.88**	**1.11–3.21**	**0.016**
Body Mass Index, mean (sd)	26.63 (5.5)	27.19 (6.1)	26.32 (5.1)	0.132	1.03	0.98–1.07	0.146
Tardive dyskinesia (AIMS score), mean (sd)	0.97 (2.2)	0.95 (2.2)	1.12 (2.4)	0.242			
Akathisia (BAS score ≥2) (%)	73 (15.9)	30 (41.1)	43 (58.9)	0.296			
Extrapyramidal symptoms (SARS score), mean (sd)	0.27 (0.4)	0.27 (0.3)	0.27 (0.4)	0.855			
Substance use disorder							
** Lifetime alcohol use disorder,** ***n*** **(%)**	**126 (28.8)**	**62 (40.8)**	**64 (22.4)**	**<0.001**	**2.58**	**1.36–4.96**	**0.031**
** Lifetime cannabis use disorder,** ***n*** **(%)**	**161 (35.2)**	**74 (45.4)**	**87 (29.5)**	**<0.001**	1.83	0.96–3.48	0.073
Treatment							
Clozapine, *n* (%)	72 (16.5)	22 (14.2)	50 (17.9)	0.324			
Long-acting antipsychotic, *n* (%)	64 (14.51)	23 (14.7)	41 (14.4)	0.918			
First-generation antipsychotic, *n* (%)	20 (4.6)	7 (4.5)	13 (4.6)	0.951			
Second-generation antipsychotic, *n* (%)	403 (92.6)	143 (92.3)	260 (92.9)	0.818			
Chlorpromazine equivalent, mean (sd)	544.31 (518.5)	613.02 (626.8)	507.47 (441.8)	0.307			
Antidepressant, *n* (%)	109 (25.1)	36 (23.2)	73 (26.1)	0.511			
Benzodiazepine, *n* (%)	99 (22.8)	35 (22.6)	64 (22.9)	0.975			
Number of psychotropic medications, mean (sd)	2.58 (1.48)	2.67 (1.6)	2.52 (1.4)	0.561			

Significant associations are in bold.

*PANSS* Positive and Negative Syndrome Scale, *AIMS* Abnormal Involuntary Movements Scale, *BAS* Barnes Akathisia Scale, *SARS* Simpson and Angus Rating Scale.

^a^aOR: adjusted odds ratio (adjusted for Age, PANSS positive score, PANSS negative score, PANSS excitation score, PANSS depressive score, PANSS disorganized score, suicide, lifetime alcohol use disorder, lifetime cannabis use disorder, Insight symptoms awareness birchwood subscore, Insight illness awareness birchwood subscore, Insight need for treatment subscore, and Body Mass Index).

Compared to individuals with follow-up data at 1 year, the “lost to follow-up” participants differed only in that they were more frequently administered long-acting antipsychotics (Table 2).

**Table 2:** Comparison between individuals with and without follow-up data.

	Patients without follow-up at 1 year (any cause)	Patients with a 1-year follow-up and a completed MARS	Univariate analysis	Multivariable analysis		
	(*N* = 616, 56%)	(*N* = 485, 44%)	*p* value	OR^a^	95% CI	*p* value
Sociodemographic characteristics						
** Sex,** **male,** ***n*** **(%)**	**439 (71.3)**	**376 (77.5)**	**0.018**	**0.53**	**0.33–0.86**	**0.010**
Age, mean (sd)	31.49 (9.3)	32.11 (10.0)	0.355			
Diagnosis, schizophrenia vs schizoaffective disorder, *n* (%)	475 (77.1)	367 (75.7)	0.575			
Clinical variables						
Age at illness onset, mean (sd)	21.18 (6.2)	21.73 (6.7)	0.173			
Illness duration (years), mean (sd)	10.15 (7.9)	10.34 (8.2)	0.869			
PANSS total score, mean (sd)	69.14 (20.3)	69.52 (17.9)	0.742			
** PANSS Positive score, mean (sd)**	**8.95 (4.6)**	**8.08 (3.7)**	**0.025**	1.04	0.97–1.11	0.179
PANSS Negative score, mean (sd)	16.43 (6.6)	15.76 (6.5)	0.108	0.99	0.95–1.03	0.524
** PANSS Excitation score, mean (sd)**	**5.85 (2.6)**	**5.29 (2.2)**	**<0.001**	1.09	0.98–1.22	0.112
PANSS Depressive score, mean (sd)	6.99 (3.1)	6.66 (3.0)	0.092	1.03	0.95–1.12	0.250
** PANSS Disorganization score, mean (sd)**	**7.71 (3.5)**	**7.24 (3.2)**	**0.034**	0.97	0.90–1.05	0.507
Insight Birchwood total score, mean (sd)	8.61 (3.0)	8.96 (2.8)	0.058	1.01	0.92–1.08	
Insight illness awareness Birchwood subscore	2.7 (1.3)	2.7 (1.4)	0.917			
** Insight symptoms awareness Birchwood subscore**	**2.83 (1.3)**	**3.01 (1.2)**	**0.011**	1.02	0.84–1.23	0.825
** Insight need for treatment subscore**	**3.10 (1)**	**3.25 (1)**	**0.023**	0.86	0.67–1.11	0.132
** Medication adherence (MARS), mean (sd)**	**6.12 (2.3)**	**6.40 (2.3)**	**0.036**	1.04	0.93–1.16	0.209
Lifetime history of suicide attempt (%)	185 (31.5)	139 (29.3)	0.452			
Body Mass Index, mean (sd)	25.96 (5.2)	26.63 (5.4)	0.052	0.96	0.92–1.01	0.143
Tardive dyskinesia (AIMS), mean (sd)	1.02 (2.4)	1.06 (2.3)	0.528			
Akathisia (Barnes score ≥2), *n* (%)	79 (14.3)	73 (15.9)	0.473			
Extrapyramidal symptoms (SARS score), mean (sd)	0.26 (0.4)	0.27 (0.4)	0.158	1.17	0.61–2.20	0.660
Substance consumption						
** Lifetime alcohol use disorder,** ***n*** **(%)**	**90 (36.4)**	**126 (28.8)**	**0.038**	1.45	0.90–2.34	0.110
Lifetime cannabis use disorder, *n* (%)	121 (34.9)	161 (35.2)	0.933			
Treatment						
Clozapine, *n* (%)	80 (16.9)	72 (16.6)	0.895			
** Long-acting antipsychotic,** ***n*** **(%)**	**95 (19.7)**	**64 (14.5)**	**0.038**	**2.26**	**1.31–4.07**	**0.004**
First-generation antipsychotic, *n* (%)	35 (7.4)	20 (4.6)	0.078	0.36	0.08–1.55	0.178
Second-generation antipsychotic, *n* (%)	422 (89.0)	403 (92.6)	0.060	0.33	0.10–1.04	0.054
Chlorpromazine equivalent	589.91 (685.7)	544.41 (518.5)	0.952			
Antidepressant, *n* (%)	97 (20.5)	109 (25.1)	0.098	0.70	0.41–1.20	0.178
Benzodiazepine, *n* (%)	119 (25.1)	99 (22.8)	0.407			
Number of psychotropic medication, mean (sd)	2.47 (1.5)	2.58 (1.5)	0.190	1.01	0.85–1.18	0.747

^a^Multivariable analysis adjusted on sex, PANSS positive, excitation, disorganization sub scores, medication adherence (MARS), body mass index, insight symptoms awareness and need for treatment sub scores, lifetime alcohol use disorder, extrapyramidal symptoms, first generation antipsychotic, second generation antipsychotic, antidepressant, number of psychotropic medication and long-acting antipsychotic.

### Medication adherence

The total mean MARS score was significantly improved at 1 year (mean difference: 0.69 ± 2.08; *t* = 7.29; *p* < 0.001, ranking from −5 to +8) as well as all of the MARS subscores (“medication adherence behavior,” 0.3 ± 1.17 (*p* < 0.001), “subjective attitudes to taking medication” 0.17 ± 1.12 (*p* < 0.001) and “subjective negative side effects,” 0.22 ± 0.83 (*p* < 0.001)).

### Clustering analysis

Hierarchical clustering analysis on the ten items of the MARS provided two identified clusters found at baseline and confirmed at the 1-year follow-up according to the AIC. A graphical representation obtained through cluster plots analysis is presented in Supplementary Fig. S1. At baseline, Cluster 1 “poor adherence” *N* = 203 (43.6%) with a mean MARS total score of 4.4 (SD = 1.5) and Cluster 2 “good adherence” *N* = 282 (56.1%) with a mean MARS total score of 8.0 (SD = 1.1). At 1-year follow-up, the two clusters solution was retained with Cluster 1 “poor adherence” *N* = 170 (35.1%) with a MARS total score of 5.1 (SD = 2.0) and Cluster 2 “good adherence” *N* = 315 (64.9%) with a MARS total score of 7.1 (SD = 1.9). The most discriminating factors between the two clusters at 1-year follow-up were Item 1 “*Do you ever forget to take your medication*?”, item 2 “*Are you careless at times about taking medication*?”, item 6 “*It is unnatural for my mind and body to be controlled by medication*”, item 9 *“I feel weird like a zombie on medication*” and item 10 “*Medication makes me feel tired and sluggish*”. The response patterns of the two adherence clusters at 1 year and the predictive importance of each item are provided in Table 3.

**Table 3:** Response pattern of the 2 adherence clusters at 12 months.

Adherence to medication at 12 months					
MARS variable	Response indicating Poor adherence	Predicator importance	Cluster 1 “Poor adherence” *N* = 170 (35.1%)	Cluster 2 “Good adherence” *N* = 315 (64.9%)	*p* value global
**1. Do you ever forget to take your medication?**	“Yes”	0.70	118 (69.4)	132 (41.9)	<0.001
**2. Are you careless at times about taking medication?**	“Yes”	0.72	101 (59.4)	104 (33.0)	<0.001
**3. When you feel better do you sometimes stop taking your medication?**	“Yes”	0.63	47 (27.6)	38 (12.1)	<0.001
**4. Sometimes if you feel worse when you take the medicine do you stop taking it**	“Yes”	0.65	70 (41.2)	62 (19.7)	<0.001
**5. I take my medication only when I am sick**	“Yes”	0.58	27 (15.9)	22 (7.0)	0.002
**6. It is unnatural for my mind and body to be controlled by medication**	“Yes”	0.78	106 (62.4)	139 (44.1)	<0.001
**7. My thoughts are clearer on medication**	“No”	0.67	97 (57.1)	102 (32.4)	<0.001
**8. By staying on medication, I can prevent getting sick**	“No”	0.59	40 (23.5)	52 (16.5)	0.059
**9. I feel weird like a zombie on medication**	“Yes”	0.73	88 (51.8)	82 (26.0)	<0.001
**10. Medication makes me feel tired and sluggish**	“Yes”	0.70	143 (84.1)	178 (56.5)	<0.001
**Total score, mean (sd)**	…	…	5.08 (2.0)	7.11 (1.9)	<0.001

*MARS* Medication Adherence Rating Scale.

Symbol: … = not applicable.

**Fig. 1:**
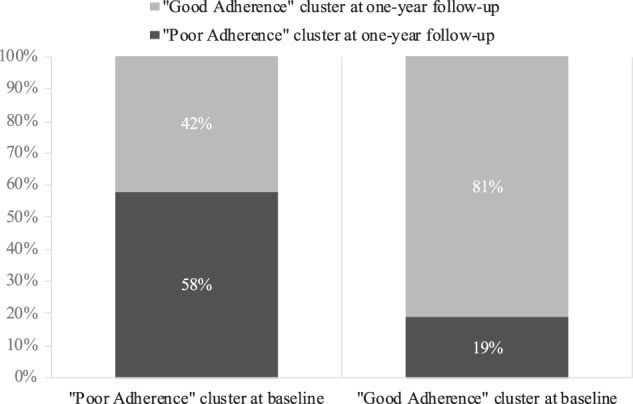
Evolution of clusters from baseline to the 1-year follow-up.

Among the 203 patients who were in the “poor adherence” cluster at baseline, 86 patients (42%) switched to the “good adherence” cluster at the 1-year follow-up and 117 patients (58%) remain in the “poor adherence” cluster. Of the 282 patients who were in the “good adherence” cluster at baseline, 53 (19%) switched to the “poor adherence” cluster at the 1-year follow-up and 229 (81%) remained in the “good adherence” cluster *χ*^2^ = 78.22, df = 1, *p* < 0.0001. The evolution of clusters from baseline to the 1-year follow-up is presented in Fig. 1.

### Baseline factors associated with the two clusters at 1-year follow-up

Univariate and multivariate models of baseline predictive factors for medication adherence at the 1-year follow-up are presented in Table 1.

The younger patients (*p* = 0.043) with higher PANSS positive scores (*p* = 0.024), PANSS depressive scores (*p* < 0.001), lower insight total score (*p* < 0.001), lower Insight needs for treatment subscore (*p* < 0.001), history of suicide attempt (*p* = 0.016) and lifetime alcohol use disorder (*p* = 0.031) had a higher risk of being classified in the “poor adherence” cluster at 1 year in multivariate analyses. These results were maintained in the inverse probability-of-censoring weighting sensitivity analysis (Table 2).

### Predictors of transition from the “Good Adherence” to “Poor Adherence” cluster

Higher depressive symptoms (aOR = 1.23, 95% CI = 1.08–1.42) and lifetime alcohol use disorder (aOR=3.36, 95% CI = 1.51–7.60) predicted the transition from the “good adherence” to the “poor adherence” cluster at 1 year in multivariate analyses.

### Predictors of staying in the “Poor Adherence” cluster

Higher depressive scores (aOR=1.16, 95% CI = 1.02–1.33) and poorer insight (aOR=0.87, 95% CI = 0.76–0.99) predicted remaining in the same cluster of “poor adherence” at 1 year in the multivariate analyses.

### Results of clinician-rated adherence (BARS)

A better clinician-rated adherence at baseline with a BARS total score of 89.37 (SD = 20.9) predicted the “good adherence” cluster at 1 year (aOR=0.98, 95% CI = 0.96–0.99). There was a significant correlation between clinician rated adherence (BARS) and patient rated adherence (MARS) (*r* = 0.37, 95% CI = 0.29–0.45, *p* < 0.001 and *r* = 0.26, 95% CI = 0.17–0.34, *p* < 0.001) respectively at baseline and follow-up).

## Discussion

Over a 1-year follow-up period, medication adherence exhibited a general improvement among a national sample of 485 patients with schizophrenia living in community settings. To achieve our primary objective, we conducted a clustering analysis using the MARS and identified key predictors of persistent medication non-adherence at 1 year: younger patients, depressive symptoms, lower insight, history of suicide attempts and alcohol use disorder. These predictive factors highlight the need to systematically screen and address these issues in order to improve adherence in schizophrenia. Moreover, the clinician ratings predicted improved adherence at 1 year (BARS), although there was only a weak correlation between clinician and patient-rated adherence.

The present study confirmed that the adherence could be clustered in 2 groups at 1 year as previously demonstrated in the same cohort at baseline [2]. Adherence is a dimension that can vary considerably from year to year in schizophrenia and, in our cohort, 42% of the initial poor adherent patients switched to the good adherent cluster at 1 year. This is an encouraging finding for interventions designed to improve adherence, such as shared medical decision making [27, 28]. However, adherence variability is also a warning sign as 19% of the patients with initially good adherence worsened at follow-up. Of note, there was no difference in adherence between lost-from-follow-up and the patients who attended followed-up, and these results were maintained through the inverse-probability weighting censuring analysis to ensure their robustness. There is therefore a low probability that these results are explained by attrition bias.

As previously demonstrated [2], the present results have confirmed that subjective negative side effects (feeling weird, tired, and sluggish, as measured by the third dimension of the MARS) were important predictors of poor adherence at 1 year. Medication adherence behavior from the first dimension of the MARS (“Do you ever forget to take your medication?”, “Are you careless at times about taking medication?”) was also predictive of poor adherence at 1 year, consistent with the fact that the BARS score also predicted adherence at 1 year. The item of the second dimension “It is unnatural for my mind and body to be controlled by medication” refers to subjective attitudes toward treatment and this item was also predictive of adherence at 1 year. Subjective and objective components of adherence are therefore both effective in predicting adherence at 1 year. Using the MARS, the three components of adherence including adherence behavior, attitudes towards anti-psychotic medication and side effects could be assessed for daily use in clinical practice. The third dimension of the BIS scale “need for treatment” was also predictive of poor adherence (but not the two other dimensions).

Impaired insight is a well-known factor associated with poor adherence [29, 30]. However, our results suggest that being aware of having schizophrenia and recognizing the functioning consequences of its symptoms do not predict future adherence. These results underscore the importance of targeting more effective educational therapy in the perspective of precision medicine. Interestingly, insight, including the “need for treatment,” has been linked to medication adherence, as demonstrated in our current data. Although this could be interpreted as a tautological phenomenon and a limitation of the multidimensional items approach employed by MARS and Birchwood, it appears particularly relevant from a clinical perspective. Patients with limited insight tend to have more concerns about taking medication and, as a result, demonstrate poorer medication adherence. Insight into the need for treatment should therefore be considered as a separate dimension of insight, and adherence-targeted interventions should focus on the need for treatment rather than on the recognition of schizophrenia and its consequences. Further investigation into both self-assessment and clinician-based assessment of insight in relation to medication adherence would be interesting. For instance, the VAGUS Insight into Psychosis Scale, encompassing self-report and clinician-rated versions, as described by Gerretsen et al. [31], could potentially yield valuable insights for future studies. Our results can be juxtaposed to those of the CATIE study in which impaired insight also predicted poor adherence at 6 months and 18 months [15].

Younger age, history of alcohol use disorder, suicide attempts, and current depression were also identified as maintaining factors for poor adherence. Suicide is the first cause of mortality in schizophrenia in young patients [32, 33] and has been associated with a poor adherence. [34, 35] Depressive disorders are highly frequent in schizophrenia, with estimates ranging from three to ten times the prevalence of the general population [36, 37]. Depressive disorders are also underdiagnosed, undertreated and frequently unremitted, and a risk factor for suicide attempt [38, 39]. Depressive symptoms have been associated with impaired adherence in schizophrenia [5, 13], but to our knowledge, this is the first time that the same association was confirmed with prospective data. The systematic assessment of comorbid depression, anxiety, and suicidality that is part of precision psychiatric evaluation moves beyond the unique focus on psychotic symptoms, and therefore allows for the prescription of antidepressants and other psychotherapeutic strategies. Approximately one in five patients with schizophrenia has a lifetime diagnosis of alcohol use disorder [40], which has been associated with resistant depression in schizophrenia [38] and poor adherence [3, 41]. Alcohol use disorder prevention, suicide prevention and treating depression are therefore priorities to be added in the care of schizophrenia. Case-managed programs may improve both suicide risk and adherence [42]. Long-acting antipsychotics is a strategy that has also been promoted to improve adherence [1, 35, 43]. However, its efficacy for medication adherence has not been confirmed although it provides the psychiatrist with the opportunity to prevent hidden non-adherence among very poorly adherent patients. In patients with suicide risk, clozapine should be prescribed according with the notion that clozapine decreases suicidal risk [44] according to international recommendations [45]. The impact of medication regimen complexity on medication adherence has been previously established [46]. Although we lack specific data on this aspect, our findings indicated that the number of psychotropic drugs, which may serve as an approximation of regimen complexity, did not show an association with medication adherence. Furthermore, we did not observe any differences in medication adherence among antipsychotics (which could also be attributed to limited statistical power within each group). A previous study has shown that higher patient cost-sharing is associated with a reduced likelihood of adhering to antipsychotic medication and a shorter duration before discontinuation of the medication [47]. Unfortunately, we were unable to investigate this question within the French context, as all patients with schizophrenia receive care under the ALD (“Affection de Longue Durée”) program and are exempt from any related charges.

Our results also confirm that the youngest patients have poorer adherence scores and are therefore the target of choice for implementing adherence-enhancing interventions [29, 30, 48, 49]. Interventions targeting medication adherence are needed at the critical early stages of the disease, which are known to be particularly at risk for relapse and suicide.

### Strengths

The multicentric, nationwide recruitment in 10 expert centers, the large battery of standardized evaluations, the longitudinal design and the sample size are strengths of the present study.

### Limits

Our assessment of adherence was limited to a subjective self-rating scale, which is susceptible to bias and is acknowledged to overestimate adherence [11]. However, obtaining accurate and cost-effective measurements to address nonadherence, a complex and multidetermined dynamic phenomenon, posing significant challenges. Direct measures such as drug plasma levels, electronic monitoring, and newly available technologies should indeed be utilized; however, they also present several shortcomings that need to be considered. With no gold standard to date, we chose the MARS due to its extensive utilization and translation in multiple languages. Furthermore, it appears more suitable for implementation in a large longitudinal cohort and for everyday clinical practice. In our results MARS and BARS are significantly correlated. However, it is important to note that MARS captures both the objective (behavioral) and subjective (attitude toward medication) aspects of adherence, whereas BARS only focuses on the behavioral component of medication adherence. Despite the large size of this national cohort study, the sample may not be representative of the overall population of patients with a schizophrenia diagnosis. While the sample was composed of outpatients referred to the various expert centers for diagnosis or treatment issues, the 10 expert centers cover a large area of the French territory and as a result integrated a wide range of socioeconomic and cultural differences. These results may only be extrapolated to patients with evolutive schizophrenia, as our sample was mean age 32 years with a mean illness duration of approximately 10 years. In summary, the FACE-SZ is representative of middle-aged patients with chronic schizophrenia consulting in the public sector in France. Other studies should be carried out in specific populations (e.g., early onset schizophrenia, first-episodes, elderly). Therapeutic alliance is also associated with adherence and this construct was not assessed in the FACE-SZ cohort [50]. The development of systematic Patient-Reported Experience Measures should address this issue [51]. Lastly, even though we employed the inverse probability-of-censoring weighting method to mitigate attrition bias, it is important to acknowledge that this method is not without limitations, and it does not eliminate all potential selection biases associated with attrition.

## Conclusions

The systematic assessment of adherence within precision psychiatry and by using validated tools provides a better understanding of important modifiable risk factors of poor adherence. Younger age, lower insight, history of suicide attempts, depressive disorders and alcohol use disorders maintain poor adherence. This latter population in particular should be targeted through literacy and educational therapy programs. Medication adherence is a dimension that can vary considerably from year to year in schizophrenia, and therefore there are significant opportunities for interventions to improve adherence. Caution is warranted, however, as almost one in five of the patients with initially good adherence worsened over the follow-up period.

### Supplementary information

Cluster Plot

## Data Availability

The data supporting the findings of this study are accessible through the Foundation Fondamental (www.fondation-fondamental.org). Please note that certain restrictions apply to the accessibility of this data, as it was utilized under license for the purposes of this study. You may request access to the data from the corresponding author with permission from Fondation Fondamental.
