# Supplementary figures and images for: Predictors of medication adherence in a large 1-year prospective cohort of individuals with schizophrenia: insights from the multicentric FACE-SZ dataset
Source: Transl Psychiatry. 2023 Nov 7;13:341. doi: 10.1038/s41398-023-02640-x (PMC10630458; doi:10.1038/s41398-023-02640-x)

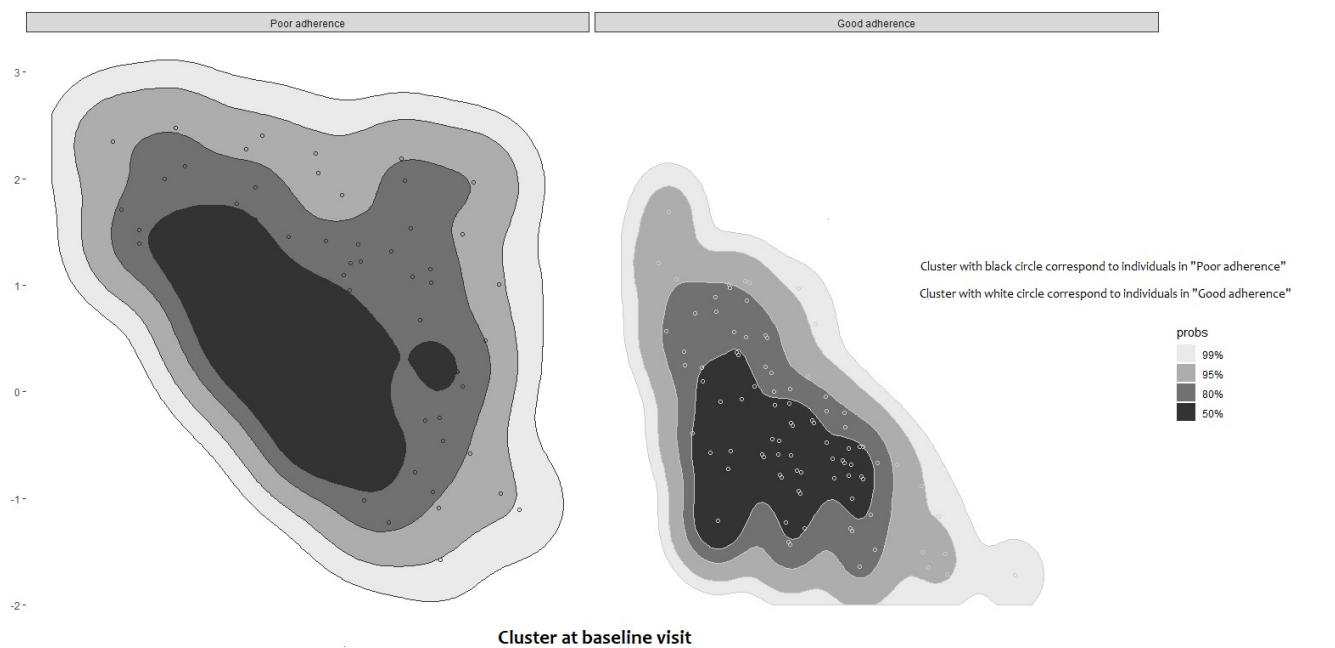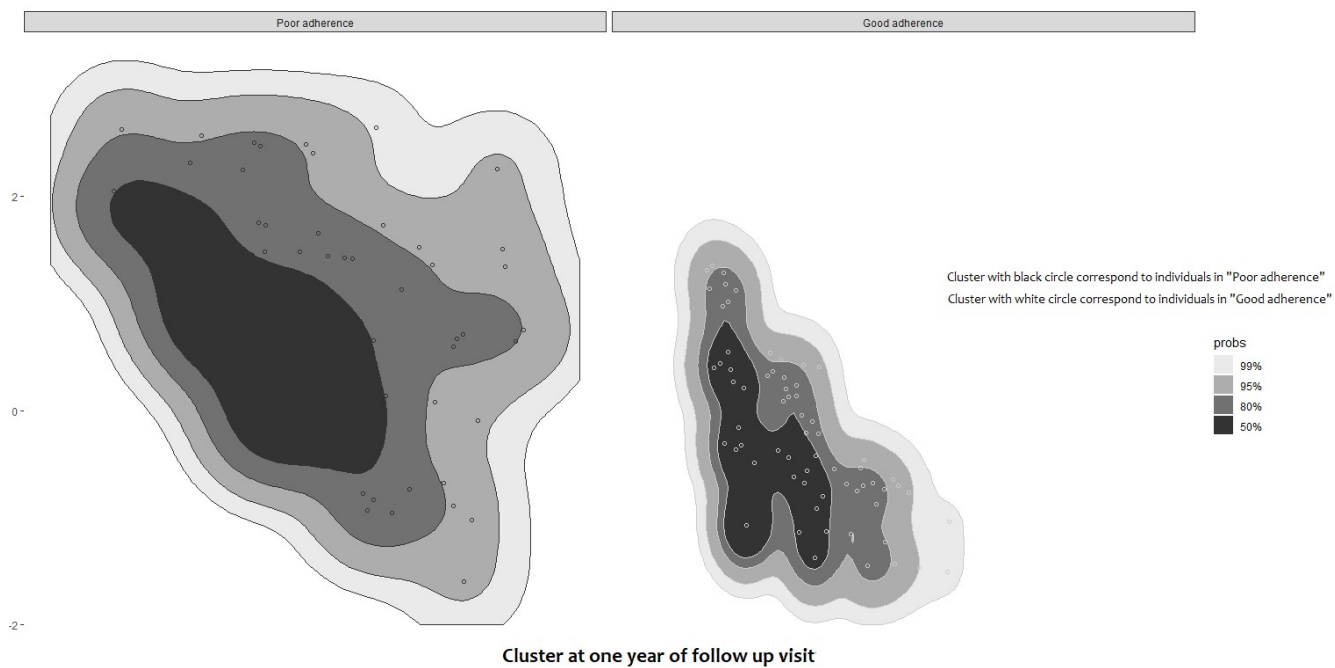

Figure S1. Cluster plots at baseline and one-year follow-up.

Supplement: Supplementary file 1 — Cluster Plot [file 41398_2023_2640_MOESM1_ESM.pdf]
